# Non-Invasive *In Vivo* Imaging and Quantification of Tumor Growth and Metastasis in Rats Using Cells Expressing Far-Red Fluorescence Protein

**DOI:** 10.1371/journal.pone.0132725

**Published:** 2015-07-17

**Authors:** Jon Christensen, Daniel Vonwil, V. Prasad Shastri

**Affiliations:** 1 Institute for Macromolecular Chemistry, University of Freiburg, 79104, Freiburg, Germany; 2 BIOSS—Centre for Biological Signalling Studies, University of Freiburg, 79104, Freiburg, Germany; Wayne State University, UNITED STATES

## Abstract

Non-invasive *in vivo* imaging is emerging as an important tool for basic and preclinical research. Near-infrared (NIR) fluorescence dyes and probes have been used for non-invasive optical imaging since in the NIR region absorption and auto fluorescence by body tissue is low, thus permitting for greater penetration depths and high signal to noise ratio. Currently, cell tracking systems rely on labeling cells prior to injection or administering probes targeting the cell population of choice right before imaging. These approaches do not enable imaging of tumor growth, as the cell label is diluted during cell division. In this study we have developed cell lines stably expressing the far-red fluorescence protein E2-Crimson, thus enabling continuous detection and quantification of tumor growth. In a xenograft rat model, we show that E2-Crimson expressing cells can be detected over a 5 week period using optical imaging. Fluorescence intensities correlated with tumor volume and weight and allowed for a reliable and robust quantification of the entire tumor compartment. Using a novel injection regime, the seeding of MDA-MB-231 breast cancer cells in the lungs in a rat model was established and verified.

## Introduction

Tumor growth and dissemination are complex processes that are not readily recapitulated *in vitro*. The tumor environment is highly complex and it presents a heterogeneous cellular composition that undergoes spatiotemporal changes, which concurrently alters the tumor microenvironment in the cellular composition. This poses many challenges with regards to tumor targeting and therapy. During the progression of a tumor, the increase in tumor volume is a combined manifestation of proliferation of tumor cells, influx of stromal cells such as mesenchymal cells, endothelial cells, tumor-associated macrophages as well as immune cells, and production of extra-cellular-matrix (ECM) [1–6]. Understanding the temporal changes to the various components within a tumor can aid in the development of improved and customized treatments and in this context, non-invasive *in vivo* methodologies to follow the molecular and cellular processes involved during tumor development are important.

In recent years, optical imaging has emerged as very useful pre-clinical tool in quantification efficacy of tumor therapies [7–10]. Among the optical imaging techniques bioluminescence (BL) has gained wide acceptance and due to its sensitivity as little as 10.000 cells can be detected *in vivo* [11]. BL signals have limited transmission through tissue due to absorption and scattering. As a result they suffer from significant attenuation with approximately a 10-fold decrease of signal intensity for every centimeter of tissue [10, 12, 13]. Signals are therefore highly surface weighted; meaning the signals from the same source becomes weaker the deeper it is located in the tissue. In addition, for BLI an imaging substrate has to be injected before imaging, so that the cells expressing the luminescent protein can enzymatically transform the substrate [10]. Therefore, in addition to depth-related limitations, BL signal is dependent on the pharmacokinetic distribution of the imaging probe in the tumor tissue. It has been shown that the peak light emission changes depending on tumor size and location [11, 14, 15]. Therefore, detection and quantification of BL signals is prone to high variability due to the aforementioned limitations. In contrast, NIR dyes and probes enable deep tissue *in vivo* imaging without the need for ionizing radiation [16, 17] and the high signal to noise ratio offers clear advantages in detecting analytes at very low concentrations [18, 19]. Consequently, imaging in the NIR window (700–1000 nm) has gained increased attention. Recent advances in instrumentation and image reconstruction algorithms now allow for tomographic projection of NIR data. Notwithstanding, to date most efforts to track cells *in vivo* have relied on covalent linkage of the dye to cells, membrane labeling with lipophilic dyes or loading the cell with fluorescence molecules, all of which pose a few limitations including the need to label the cells immediately prior to injection, dilution of the label during cell proliferation and loss of label due to hydrolysis *in vivo*. Loss of the label over time can impede the accurate determination of cell number and therefore long-term monitoring. Cells stably expressing fluorescence proteins on the other hand can provide an advantage over cell labeling systems and BLI; as the fluorescent label is integrated into the genome and as result the fluorescence is retained in the daughter cells making it possible to follow cell proliferation. Additionally, since fluorescence proteins do not require any co-injection of imaging agents, the fluorescence signal can be more accurately assigned to a location in the animal subject and can be followed over time.

Recently, we have validated a fluorescence trans-illumination imaging modality in rats and demonstrated the ability to assign quantitative fluorescent information with anatomical accuracy by co-registering the fluorescence volumetric reconstructions with microcomputer tomography (μCT) reconstructed volumes [20]. As a pre-clinical model, rats offer many advantages over mouse in studying tumor metastasis as surgical manipulation of the skeletal compartment is more readily achieved and also larger tumor burdens may be attained [21–23]. Additionally, drug pharmacokinetics in rat subjects allow more realistic circulation kinetics and partitioning as well as more frequent blood sampling [24]. Building on these findings in this study, we transduced MDA-MB-231 to stably express E2-Crimson, a far-red fluorescence protein with excitation (Ex_max_ 611 nm) and emission (Em_max_ 646 nm) that is suitable for imaging in the NIR window [25]. Using these cells we visualized and quantified the formation of mesoscopic subcutaneous tumor burden in the flank of rat subjects over a period of 5 weeks. Fluorescent intensities correlated with tumor weight and volume as determined using calipers (Pearson correlation 0.9490 and 0.8667 respectively). By combining an agent that can target blood vessels and angiogenesis, with E2-Crimson expressing tumors, the concurrent visualization of vasculature associated with a tumor was accomplished in live subjects. Finally, by combining fluorescence tomography with μCT we demonstrate for the first time the seeding of MDA-MB-231 cells to lung tissue upon tail vein administration in rats and thus laying the foundation for the utilization of this model in tumor biology.

## Materials and Methods

### Cell lines

The epithelial cell lines MDA-MB-231, HEK293, A594 and HeLa were cultured in Dulbecco’s modified Eagle essential medium (DMEM) Glutamax containing 10% fetal calf serum (FCS) and supplemented with penicillin and streptomycin at 37°C and 5% CO_2_ in a humidified incubator. The cell lines were provided by the Centre for Biological Signalling Studies (BIOSS) and were genotyped and verified by Labor für DNA Analytik (Freiburg, Germany).

### Lentiviral production and transduction

The fluorescence protein E2-Crimson and CMV promoter was amplified from the pE2-Crimson vector (Takara Bio, Germany) (forward primer CGGAATTCATGGAGCACTG and reverse primer GCGAATTCCTACTGGAACAGGT) and cloned into the lentiviral shRNA vector pGIPZ (Fisher Scientific, Germany). The TurboGFP and associated CMV promoter was exchanged by using the XbaI and NotI restriction sites. Lentiviral particles were produced in HEK293 cells, by co-transfecting lentiviral vector and packaging vectors using polyethylenimine (Mw 25.000, Sigma, Germany) as the transfection reagent. For transfection, 30 μg of DNA (4:3:1 of transfer vector, packaging coding vector (pCMV-dR8.74) and envelope coding vector (pMD2.G)) was diluted in 250 μL Opti-MEM (Invitrogen, Germany) and 11.25 μL of polyethylenimine (1 mg/mL) was added to the solution and the resulting mixture was incubated for 1 hour at room temperature prior to adding to HEK293 cells. The medium was changed after 16 hours to DMEM with 10% FCS and 40 and 64 hours after transfection the viral supernatants were collected and filtered through a sterile 0.45 μm syringe filter (Millipore, Germany). Viral particles were added to target cells (MDA-MB-231 and HeLa) supplemented with 5 μg/mL polybrene (Sigma, Germany). 3 days after transduction, infected cells were selected by adding 5 μg/mL puromycin (Sigma, Germany) to the culture medium.

### Migration studies

Cells were deprived of serum for 24 hours prior to commencing the migration. Cells were harvested and washed two times with PBS and re-suspended in migration buffer (DMEM 1% BSA). Migration assay was carried out in a 24-well plate with inserts having a pore size of 8 μm (VWR, Germany). 500μL DMEM with 10% FBS was pipetted into the chamber and the insert was placed following which 25.000 cells suspended in 150μL migration buffer were seeded on top of the insert. Cells were allowed to migrate for 15 hours at 37°C, after which period the inserts were washed on both sides with ice cold PBS and non-migrated cells were removed with a cotton swab and the inserts were dried and stained with Diff Quick (Siemens Healthcare, Germany). 5 random fields were counted per insert at a 400X magnification. Data is represented as number of cells per 5 high-powered fields (5HPF).

### Cell proliferation

Cells were seeded at a density of 20.000 cells/well in 6 well plates and the growth medium was renewed every 3 days. Cells were trypsinized, centrifuged at 500 g, resuspended in PBS and counted using a hemocytometer (VWR, USA).

### Validation of E2-Crimson expressing cells for fluorescence imaging

Optical phantoms (Perkin-Elmer) were loaded with 100 μL of a cell suspension in PBS and scanned using the fluorescence molecular tomography system FMT2500 (Perkin-Elmer). The total cell number in the phantom ranged from 1×10^4^ to 5×10^6^.

### Xenograft rat model

Female athymic nude mutant rat (RH-Foxn1^rnu^) 5–6 weeks old, (average weight 98.0 g ± 17.2 g) were procured from Harlan (Germany) and housed and handled in accordance with good animal practice as defined by FELASA and the national animal welfare body GV-SOLAS. Animals were kept on C1039 non-fluorescent chow (Altromin, Germany) diet and water *ad libitum*. All animals were monitored daily for tumor related symptoms and weighed two times per week. All experiments were done with the approval of the animal welfare committees of the State of Baden Württemberg and University of Freiburg (Regierungspräsidium Freiburg Az.: 35/918.581/G-13/80).

A single tumor burden per animal was created by subcutaneous (s.c.) injection of 10^7^ MDA-MB-231 wild-type or MDA-MB-231 E2-Crimon expressing cells suspended in 100 μL of Matrigel (BD Biosciences, Germany) solution in PBS (1:1 v/v). The growth of the tumor was monitored using FT and calipers three times a week. Tumor volume was estimated by using the formula V = D*d^2^ / 2, where D is the long axis and d the short axis of the tumor. The animals were monitored continuously for signs of distress or pain by trained personnel and in accordance with the university veterinarian’s German federal guidelines for the care and maintenance of laboratory animals. This included daily observation of behavior/appearance and biweekly measurements of body weight and tumor size. During this study none of the animals displayed symptoms of suffering or reached the termination criteria (tumor size > 30 mm, weight loss > 20%) as defined by society of laboratory animal science GV-SOLAS. Rats were sacrificed after 5 weeks and the tumors were surgically resected, weighed, and the tissue was prepared for scanning with FT, flow cytometry and immunohistochemistry (IHC). For imaging of the tumor vasculature, anesthetized animals were injected with 0.096 nmol of AngioSense-750 (Perkin Elmer) per gram body weight via the tail vein and 24 hours post injection animals were scanned using FT.

### Induction of lung tumors

Lung seeding was accomplished by three sequential injections of MDA-MB-231 E2-Crimson expressing cells via the tail vein (3×10^6^ in 400 μL) over a 5-day period starting with day 0, day 1 and day 4. Animals were scanned three times a week using FT and once a week with μCT. Rats were sacrificed after 6 weeks and the thoracic cavity opened and scanned with FT. Lung, liver, heart, spleen and kidneys were excised and imaged with FT *ex vivo*.

### Fluorescence tomography (FT)

Animals were imaged as previously described [20]. Animals were anesthetized with a mixture of isofluorane and oxygen. The anesthetized animals were placed in a multimodal imaging cartridge that allows for co-registration of FT and μCT images. After inserting the imaging cartridge in the FMT2500 scanner (Perkin Elmer) a planar reflectance imaged was captured to identify fluorescence sources. A grid was placed around the fluorescence source and 3D scans were carried out. The resulting 3D fluorescence volumes were reconstructed using TrueQuant software (Perkin Elmer). For quantification of fluorescence intensities and volume size TrueQuant analysis software was used. A region of interest (ROI) was created around the reconstructed volume and fluorescence intensity and volume of fluorescence source as mm^3^. Fluorescence intensity here represents the absolute fluorescence as computed by TrueQuant.

### μCT imaging

Animals were scanned using a SkyScan 1178 (SkyScan, Belgium; version 1.3) high speed *in vivo* micro-CT imager over an angle of 180° with rotation steps of 1° at a resolution of 83 μm and a field of view of 86 mm. Each image was acquired at 65 kV and 615 μA as an average of three frames. Using the NRecon module (SkyScan 2010, v.6.3.3), the data sets underwent post-alignment, beam-hardening correction, as well as ring-artifact reduction (parameter set with the integrated fine-tuning tool) and the 3D reconstructions were exported in a DICOM format.

### Image co-registration

The μCT volumes (isometric voxel size: 0.0844 μm; 908×428 pix, 500–1,200 slices) were imported into AMIDE (64-bit v.1.0.4), corrected for axial rotations and manually stitched. FMT volumes were also imported into AMIDE and rigidly co-registered with the micro-CT volumes based on the fiducial marks. The co-registered volumes were imported into Osirix (v. 5.5.1, 64-bit). In Osirx, the volumes were synchronized, fused and visualized using the 3D volume rendering.

### Flow cytometry

Part of the dissected tumors were finely minced with a scalpel and washed with PBS. The tissue was then digested with collagenase II (Invitrogen, Germany) for 1 hour at 37°C and passed through 40 μm mesh (Millipore, Germany). After washing with PBS the cells were fixed with 4% paraformaldehyde (Sigma, Germany) for 10 minutes at 4°C and analyzed by flow cytometry (Gallios, Beckman Coulter, Germany).

### Immunohistochemistry

Tumor samples were snap frozen in OCT and stored at -80°C until processing. Tissue blocks were sectioned into 8 μm slices on a cryostat (Zeiss, Germany). Sections were fixed in 4% paraformaldehyde for 10 minutes at 4°C and washed two times with PBS. Next sections were incubated in blocking buffer (PBS Tween 0.1% and 1% goat serum) for 1 hour at room temperature and subsequently incubated with primary antibody diluted in blocking buffer (1:20) overnight at 4°C. Primary antibodies used were: polyclonal rabbit anti-human Ki-67 (Ab15580 Abcam, Germany, Antibody Registry identifier AB_443209), polyclonal rabbit anti-human CD31 (Ab28364 Abcam, Germany, Antibody Registry identifier AB_726362) and monoclonal mouse anti-human nuclei (HuNu) (MAB1281 Millipore, Germany, Antibody Registry identifier AB_94090). The following day, sections were washed three times for 5 minutes with PBS and secondary antibody was added to the sections and incubated for 1 hour at room temperature. The sections were then washed in PBS 0.1% Tween three times for 5 minutes and then mounted with Vectashield (Biozol, Germany) and imaged using a fluorescence microscope (Zeiss Cell Observer-Z1, Germany).

## Results and Discussion

The purpose of this study was to establish a system that allowed continuous, non-invasive monitoring of tumor growth and detection of occult tumors in rats *in vivo* using NIR-fluorescence tomography. Currently, the growth of a tumor in a pre-clinical rodent model is assessed by palpation, and semi-qualitative methods are applied to determine tumor volume [26]. Since the tumor volume data is typically gathered at the end of the study, very little insight into the temporal changes in tumor volume is available. The absence of real-time information on the cellular makeup of the tumor, and true tumor volume and dimensions precludes the gathering of clinically relevant information relating tumor stage to responsiveness to therapy. Although, an E2-crimson, enzyme firefly luciferase, herpes simplex virus-1 thymidine kinase, triple modality reporter system has been recently described by Tsien an co-workers in mice subjects, the NIR reporter was not used to gather information on tumor cellularity or volume [27]. Therefore, the primary objectives of this study were to develop and validate E2-Crimson expressing cancer cells in order to image cellularity and tumor volume in a rat subject.

### *In vitro* validation of E2-Crimson expression in epithelial tumor cells

**Fig 1:**
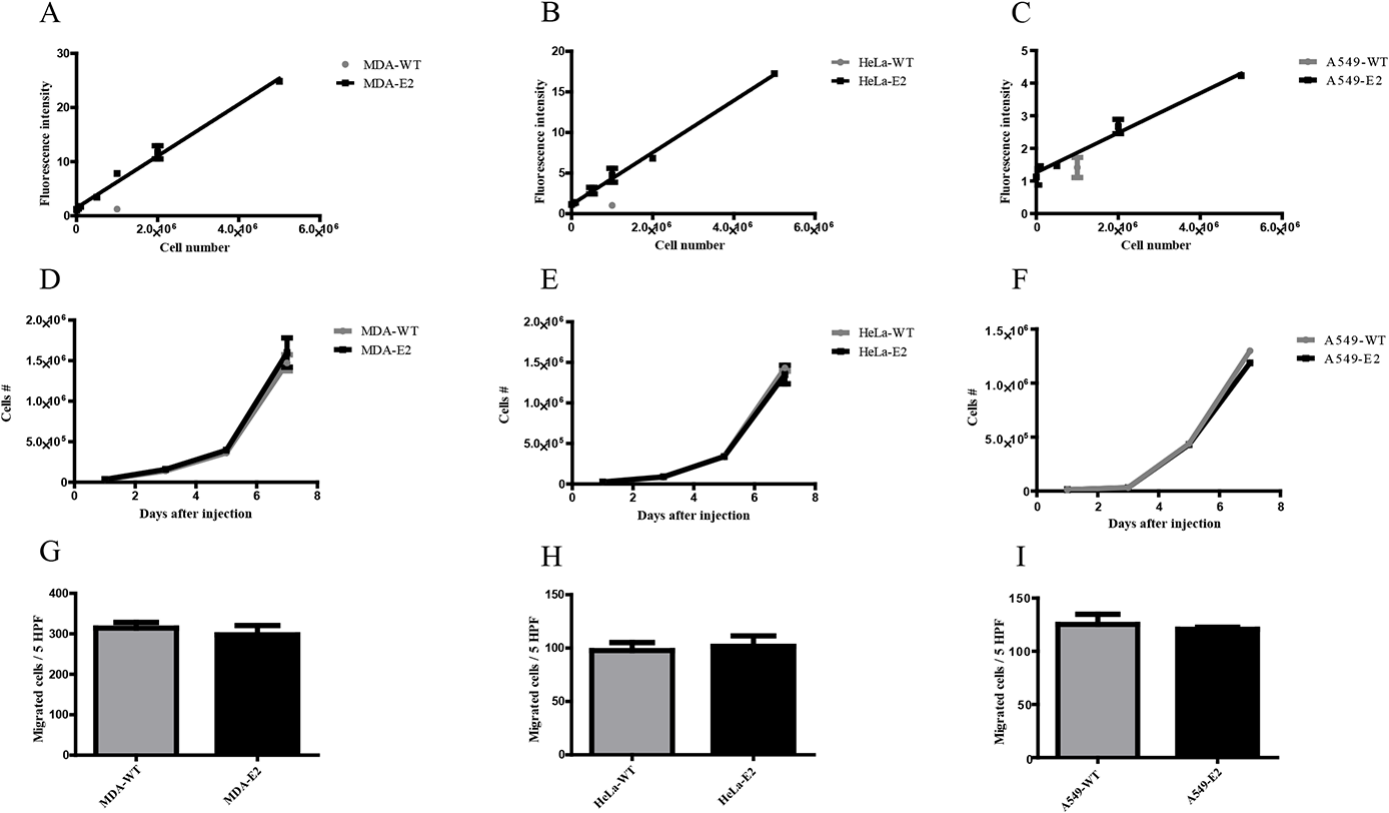
*In vitro* analysis of cell lines expressing E2-Crimson. The fluorescence intensity of various amounts of (A) MDA-E2, (B) HeLa-E2 and (C) A594-E2 cells, were measured in optical phantoms using FT. Non transduced parental cell lines (denoted with WT) were only measured at a concentration of 10^6^ cells. (D-F) Cell viability and (G-I) migratory potential was compared between E2-Crimson expressing cells and the wild-type parental cell lines. All data is represented as mean ± standard deviation. Significance was measured by Students t test. n = 3.

As the first step, cancer cell lines from cervical, lung and breast epithelial origins (HeLa, A594 and MDA-MB-231, respectively) were engineered to stably express E2-Crimson (from here on E2-Crimson expressing cells are denoted HeLa-E2, A594-E2 and MDA-E2 and parental wild type cell lines are denoted as HeLa-WT, A594-WT and MDA-WT). The detection of E2-Crimson expressing cell lines was carried out by placing them in an optical phantom to emulate the *in vivo* setting. The fluorescence signals from HeLa-E2, A594-E2 and MDA-E2 showed linear correlation with concentration, which is essential for quantification of cell number in a tumor volume (Fig 1A–1C). It has been reported in the literature, that expression of fluorescent proteins can alter cell behavior [28]. Importantly, E2-Crimson expression did not alter the migratory or proliferative capacity of the cells (Fig 1D–1I). Although, differences in fluorescence intensity between cell lines was observed, which is most likely due to differences in CMV promoter activity depending on the cell line and copy number integration; this did not preclude the utilization of these cells in future studies.

### MDA-E2 cells show tumor forming capacity *in vivo*

**Fig 2:**
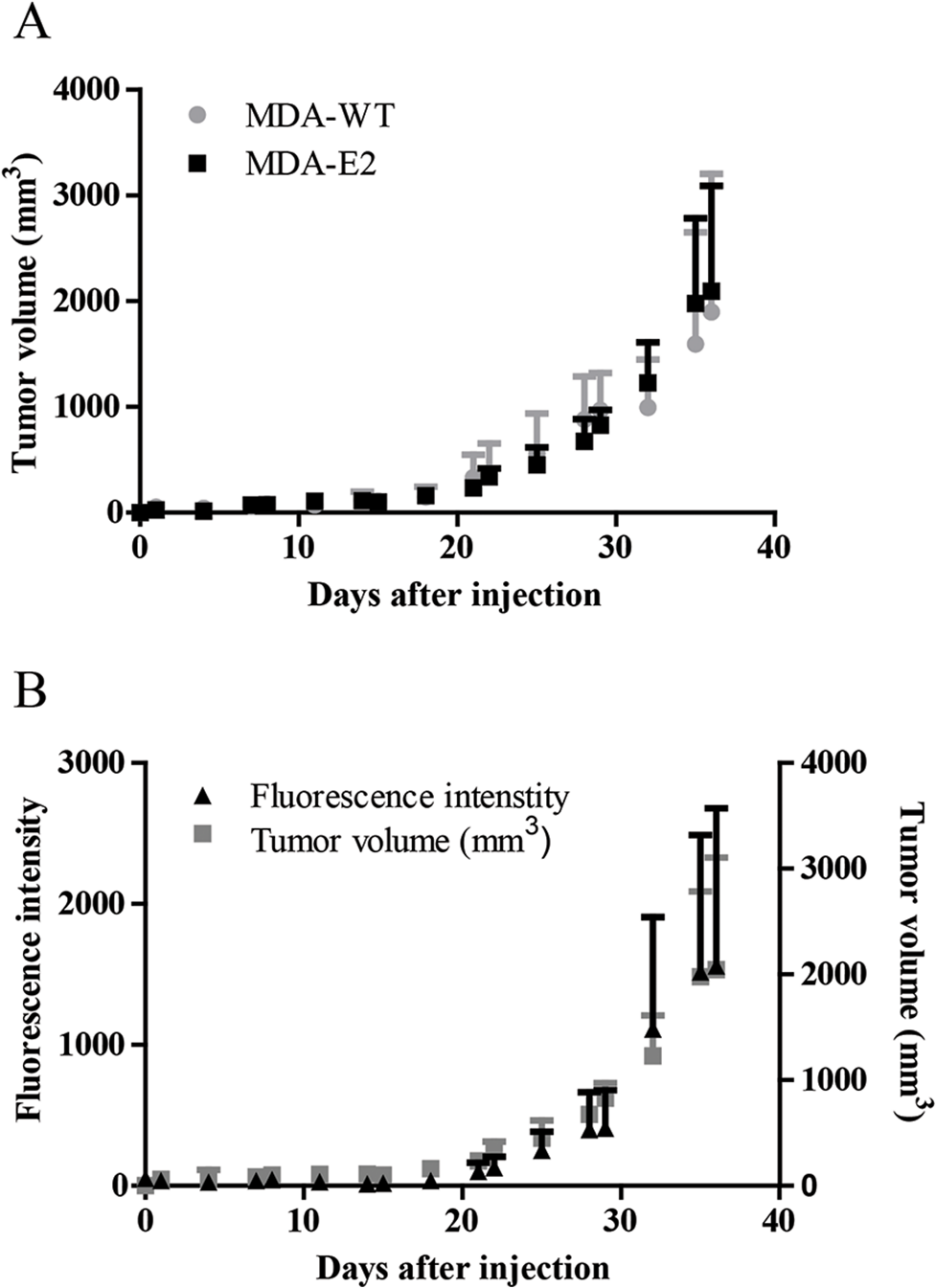
Long-term detection of MDA-E2 cells *in vivo*. (A) MDA-E2 or MDA-WT tumor cells were injected in the flank of nude rats and the tumor size was measured using calipers. n = 5–6. (B) Rats bearing MDA-E2 tumors were scanned with FT. Left axis fluorescence intensity (black triangles) and right axis tumor volume (grey squares) measured using calipers. n = 6. All data is represented as mean ± standard deviation.

Due to its extensive use in establishing tumors in mice and rat models [29, 30] and its metastatic potential [31–33] the human breast cancer cell line MDA-MB-231 was chosen for further *in vivo* studies. We first examined if MDA-E2 cells were capable of establishing tumors in a rat model by comparing the *in vivo* growth of MDA-WT and MDA-E2 tumors (Fig 2A). We observed no differences in tumor size over the course of 5 weeks between MDA-E2 and MDA-WT and similar take-rates in both cell systems (6/8 and 5/8 for MDA-E2 and MDA-WT, respectively), suggesting that E2-Crimson expression did not alter the tumor forming capacity and growth of MDA-MB-231 cells *in vivo*.

### MDA-E2 tumors can be detected *in vivo* using fluorescence imaging

We next investigated if the fluorescence signal from MDA-E2 tumors could be detected *in vivo* and allocated to the tumor. FT scans revealed that the *in vivo* fluorescence signals were located superficially on the back of the animals and this overlapped qualitatively with the palpable tumor mass (S1A Fig). *Ex vivo* imaging of explanted tumors confirmed that the fluorescence originated from the tumor mass. Thus, the ROI´s delineated in the FT scans accurately identified fluorescence signals that were assignable only to the tumors (S1B Fig) with no contribution from background fluorescence or other organs (S1C Fig). In order to further verify the participation of MDA-E2 cells in the formation of tumors; tumor samples were analyzed using flow cytometry. This showed that approximately 95% of the tumors were composed of E2-Crimson positive cells (S2A Fig). This was further confirmed by immunofluorescence microscopy of tumor sections, which showed that the majority of the cells in the tumor were indeed of human origin (S2B Fig). Interestingly, these tumors were undetectable using μCT, thereby exalting the advantage of using highly sensitive fluorescence-based detection in tumor biology.

### MDA-E2 tumors enable continuous monitoring of *in vivo* tumor growth

Cells expressing fluorescence protein can facilitate imaging and quantification of tumors, as it is not necessary to inject probes or pre-label cells with contrast agent. Furthermore, continuous imaging of all injected tumor cells and their progeny can be carried out regardless of phenotype thus allowing for accurate quantification of relative tumor load. The fluorescence intensities associated with the E2-Crimson expressing tumors mirrored the tumor volume over a 5 week period (Fig 2B and S3 Fig). Correlation analysis between fluorescence intensity and tumor volume over the entire duration of the experiment showed a highly significant correlation (Pearson = 0.9469, p-value = 0.0011), thus establishing the ability to reliably measure tumor load and growth using cells expressing far-red fluorescence proteins.

### Fluorescence-based quantification can substitute traditional tumor end-point measurements

Since this study is the first attempt to correlate E2-Crimson fluorescence with tumor progression, a validation of the fluorescent data with routinely used endpoint measurements in cancer biology namely tumor volume and weight was undertaken. The following parameter were compared: 1) *in vivo* fluorescence intensity and tumor volume (measured by calipers), 2) *in vivo* fluorescence intensity and tumor weight, 3) tumor volume (estimated by FT) and tumor volume (measured by calipers), and 4) tumor volume (estimated by FT) and tumor weight (Table 1). Analysis of fluorescence intensity originating from the tumor cells showed strong correlation with tumor volume and weight (Table 1, columns 1 and 2). In spite of the limited volumetric resolution of the FT scanner (1 mm^3^ voxel) the reconstructed fluorescence volumes correlated well with tumor volumes measured by calipers, but showed weaker correlation with tumor weight (Table 1, column 3 and 4). Taken together this data demonstrates that E2-Crimson expressing cells can be detected *in vivo*, are suitable for tumor engraftment, and offer an non-invasive complementary methodology to follow tumor progression in real-time, while providing distinct advantages over purely end-point based analyses.

**Table 1:** Correlation analysis of fluorescence intensities and tumor volume computed from FT scans compared to standard endpoint measurements tumor size (estimated from calipers) and weight.

	FT. Fluorescence intensity		FT. Volume (mm^3^)	
	1. Tumor Volume (mm^3^)	2. Tumor Weight (mg)	3. Tumor Volume (mm^3^)	4. Tumor Weight (mg)
Pearson correlation coefficient	0.8667	0.9490	0.8239	0.7215
p-value	0.0132	0.0009	0.0271	0.0823

### Delineation of cell and ECM contribution in tumor progression

**Fig 3:**
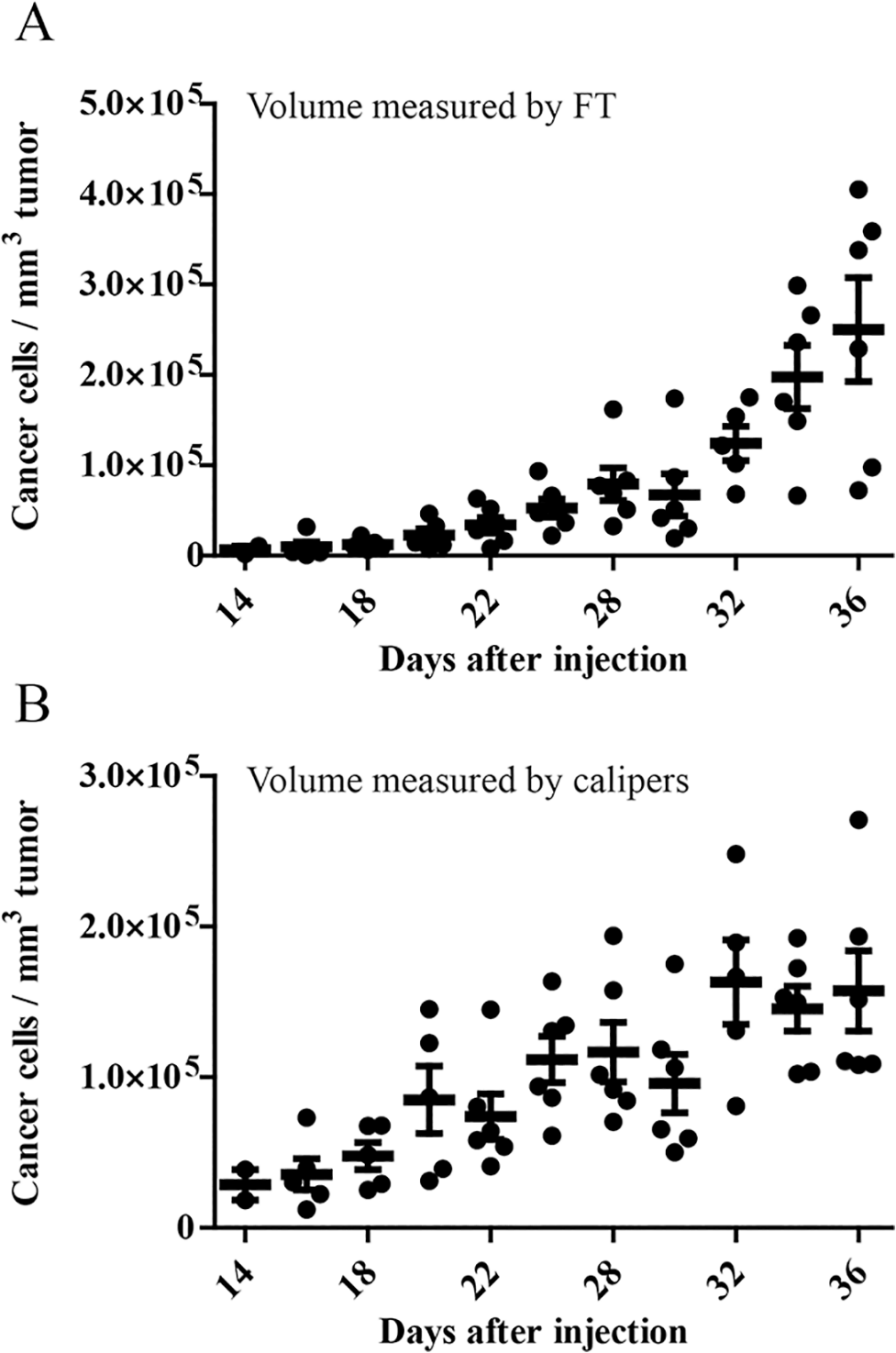
Determination of the concentration of cancer cells in the tumor during tumor growth. Fluorescence intensities of MDA-E2 tumors were converted to cell number using linear regression from (Fig 1A). The total calculated cell number was divided with the corresponding tumor volume measured by (A) FT volumetric reconstructions or (B) calipers, for each animal. Black circles correspond to single animals. All data is represented as mean ± standard deviation. n = 6.

Tumor volume and weight are routinely used in preclinical and clinical settings to determine tumor progression and treatment efficacy. However, these values do not truly reflect the tumor composition. Different amounts of ECM, stromal and tumor cells can be present in a tumor, depending on the location of the tumor engraftment and stage of the tumor. To understand the relationship between tumor volume and cell number during tumor development, we determined the volumetric density of cells based on the fluorescence intensity (Fig 1A) and volumes estimated from FT or caliper measurements (Fig 3A and 3B). It can be clearly seen that the volumetric density of cells increases over time with the increase in tumor mass. However, a surprising finding of this study was that during the earlier phase of tumor development up to 4 weeks, ECM production, rather than increase in cell numbers was responsible for the increased tumor volume. In contrast, after 4 weeks until the end point of the study, a dramatic increase in tumor cell number was observed, with no significant proportional changes to tumor volume. This study for the first time provides visual and quantitative evidence for biphasic growth behavior in tumors, where ECM deposition is followed by a proliferative phase, and has implications for the choice of treatment and dosing.

### E2-Crimson expressing tumors are permissible to simultaneous imaging of tumor mass and tumor associated vasculature

**Fig 4:**
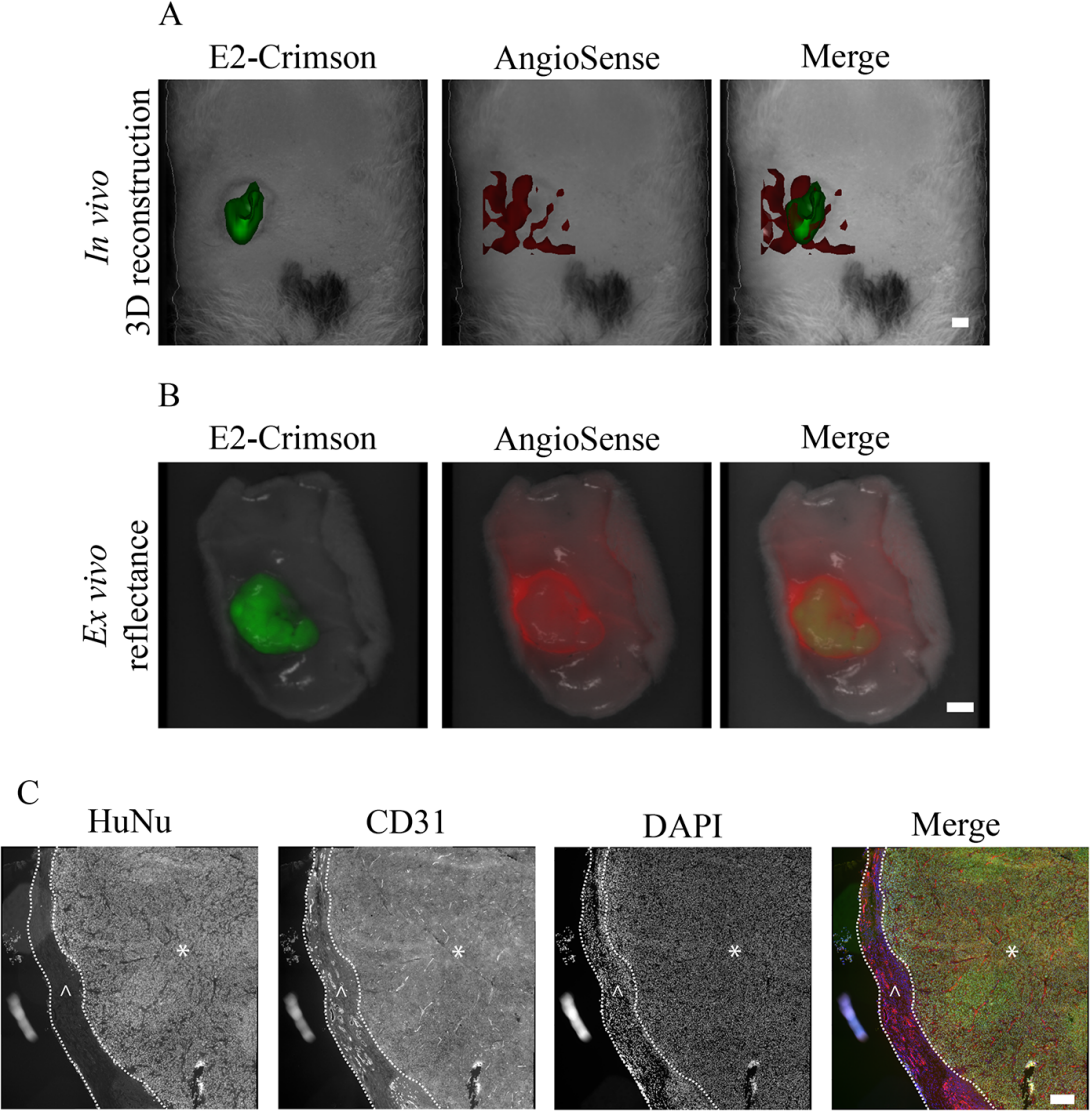
*In vivo* imaging and visualization of tumor mass and vasculature. (A) FT 3D reconstructed volumes of a rat bearing a MDA-E2 tumor (green) and the associated vasculature (red) visualized using AngioSense. Bar indicates 4.1 μm. (B) *Ex vivo* reflectance images of the corresponding tumor in (A). Bar indicates 4.1 μm. (C) Representative histological images of the tumor in (A) and (B) stained with antibodies against CD31 (red) and human nuclei (green). The nuclei were stained with DAPI (blue). The tumor mass is marked with (*) and the surrounding vascularized host stroma with (^). Bar indicates 100 μm.

FT enables the 3-D rendering of fluorescence signals, which in turn allows for the mesoscopic visualization of biological processes and tissues *in vivo*. Tumor vascularization remains a valuable therapeutic target, and therefore, visualization of the tumor and its associated vasculature is of significant interest. By administering AngioSense, a probe that targets neo-vasculature to rats bearing subcutaneous MDA-E2 tumors, we were able to simultaneously visualize the tumor and the associated vasculature and assign it to the appropriate tumor compartment (Fig 4A). In doing so we made another interesting observation that the tumor itself appeared poorly vascularized, and the majority of the fluorescence signal from the AngioSense probe was located adjacent to the tumor. This was further verified by the *ex vivo* imaging of the excised tumor and the surrounding tissue (Fig 4B) and histological analysis which showed that the tumors were surrounded by a layer of host stromal cells, and this outer layer was heavily vascularized compared to the cancerous core (Fig 4C). It should be noted that proliferation of tumor cells was observed throughout the sample cross-section suggesting a sufficient nutritional supply (S4 Fig). This accurate spatial assignment of a cellular process to a designated compartment within the tumor was possible because the E2-Crimson expression within the tumor enabled the delineation of the fluorescence signal associated with the vasculature from the tumor volume.

### Seeding of MDA-E2 cells to the lungs in rat

**Fig 5:**
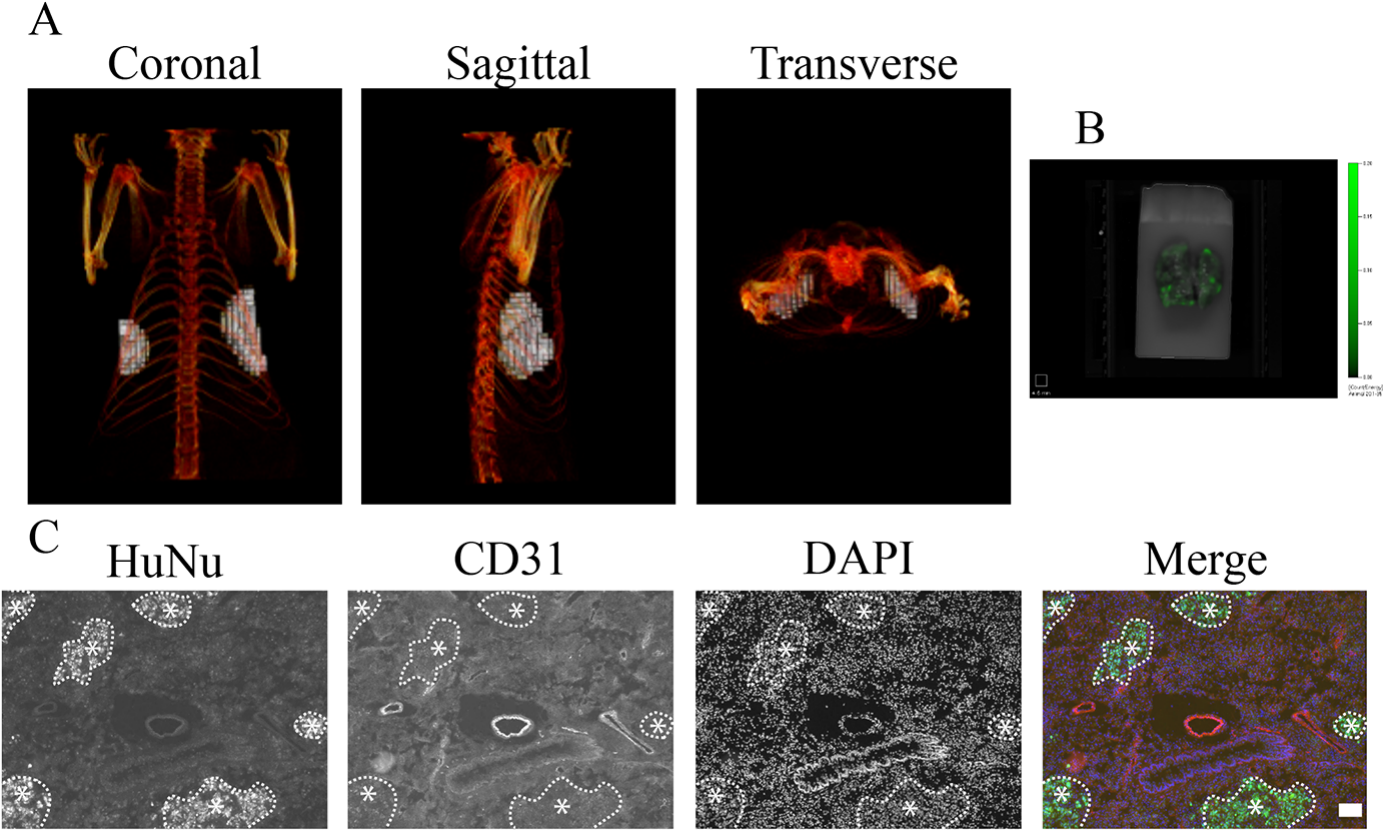
*In vivo* detection of tumors in the lung. (A) Co-registration of FT and μCT data sets 36 days after tumor cell injection. Light gray indicates fluorescence signal detected by FT, skeletal structures were visualized by μCT and are colored red. (B) E*x vivo* reflectance image of the corresponding lung. (C) Representative histological image of the lung tissue with antibodies against CD31 (red) and human nuclei (green). The nuclei were stained with DAPI (blue). Clusters of human tumor cells are marked with (*).Bar indicates 100 μm.

Since NIR fluorescence signals residing deep within tissue can be detected with minimal loss of signal intensity, E2-Crimson expressing cells might therefore be suitable for detection of intra-organ metastasis. We had recently reported that allocation of reconstructed FT volumes with a great deal of anatomically accuracy can be achieved by co-registration of FT data sets with μCT [20]. As stated earlier, rat models offer several advantages over mice; however, metastatic models of epithelial tumors in rats are not prevalent. We therefore set up an experimental model, wherein seeding of tumor cells to the lung could be established by sequential tail-vein injections of MDA-E2 cells. The occurrence of tumor nodules was confirmed by mapping the fluorescence of MDA-E2 (Fig 5A), and by combining FT volumes with μCT imaging modality the fluorescence source was accurately assigned to the left and right side of the thoracic compartment, corresponding to the anatomical location of the lungs (Fig 5A). While the presence of fluorescence throughout the lung was further confirmed by *ex vivo* imaging of the lung (Fig 5B), the allocation of the nodules using the reconstructed FT volumes to the lung tissue was explicitly verified by histological examination of the lung nodules which confirmed that the fluorescence source found in the lungs originated from injected MDA-E2 cells (Fig 5C).

## Conclusion

Functional imaging is becoming an invaluable tool in preclinical oncology. In this context NIR FT offers many advantages including continuous monitoring and 3D visualization of tissues. Using MDA-MB-231 stably expressing the far-red fluorescence protein E2-Crimson, the growth of tumors in rats and its associated cellularity was followed over time using FT and ability to visualize tumor cell seeding to the lungs was demonstrated. Additionally, FT derived tumor volumes were correlated to tumor volumes determined using traditional techniques. The ability to visualize cellularity within tumors over time by combing E2 Crimson expression with FT led to a novel finding that the early phase of tumor growth was dominated by ECM production followed by cancer cell proliferation. Overall E2-Crimson expressing cells in combination with fluorescence tomography offer a powerful tool for preclinical research. However, further studies in syngeneic tumor models need to be undertaken and the immunogenicity of E2 Crimson expressing cells ascertained, even though Puissant *et al*. have reported no immune response to E2 Crimson expressing cells in myeloid leukemia models [34].

## Supporting Information

S1 Fig *In vivo* MDA-E2 FT signal localizes to the tumor. Representative image sequence of a MDA-E2 tumor bearing animal. (A) I*n vivo* reflectance images showing fluorescence (green) overlapping with a visible and palpable tumor. (B) E*x vivo* reflectance scan of the corresponding tumor and surrounding skin. (C) Reflectance image of the abdominal region where the tumor was situated.
(TIF)

S2 Fig Validation of MDA-E2 tumors cellular composition. (A) Flow cytometry histogram of cells from dissociated tumor tissue from MDA-WT (black line) and MDA-E2 (gray line). (B) Representative histological image of MDA-E2 and MDA-WT tumors stained with antibody against human nuclei (green). The nuclei were stained with DAPI (blue).
(TIF)

S3 Fig Longitudinal image sequence of MDA-E2 tumors. (A) Representative longitudinal image sequence of an animal bearing a MDA-E2 tumor. Images were taken at indicated times using FT.
(TIF)

S4 Fig Histological features of MDA-E2 tumors. Representative histological images from MDA-E2 tumors. (A) MDA-E2 tumor stained with antibodies against CD31 (red) and human nuclei (green). The nuclei were stained with DAPI (blue). (B) MDA-E2 tumor stained with antibodies against Ki67 (red) and human nuclei (green). The nuclei were stained with DAPI (blue).
(TIF)
