# Supplementary figures and images for: Non-Invasive In Vivo Imaging and Quantification of Tumor Growth and Metastasis in Rats Using Cells Expressing Far-Red Fluorescence Protein
Source: PLoS One. 2015 Jul 17;10(7):e0132725. doi: 10.1371/journal.pone.0132725 (PMC4505884; doi:10.1371/journal.pone.0132725)

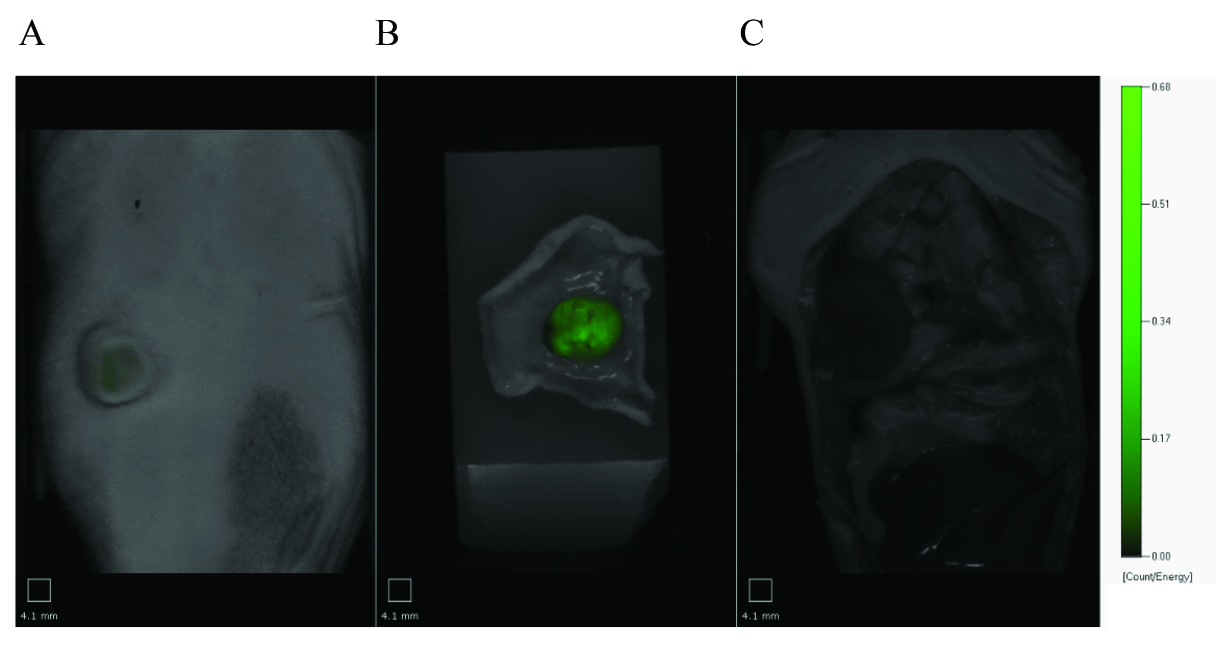

Supplement: S1 Fig — Representative image sequence of a MDA-E2 tumor bearing animal. (A) In vivo reflectance images showing fluorescence (green) overlapping with a visible and palpable tumor. (B) Ex vivo reflectance scan of the corresponding tumor and surrounding skin. (C) Reflectance image of the abdominal region where the tumor was situated. (TIF) [file pone.0132725.s001.tif]

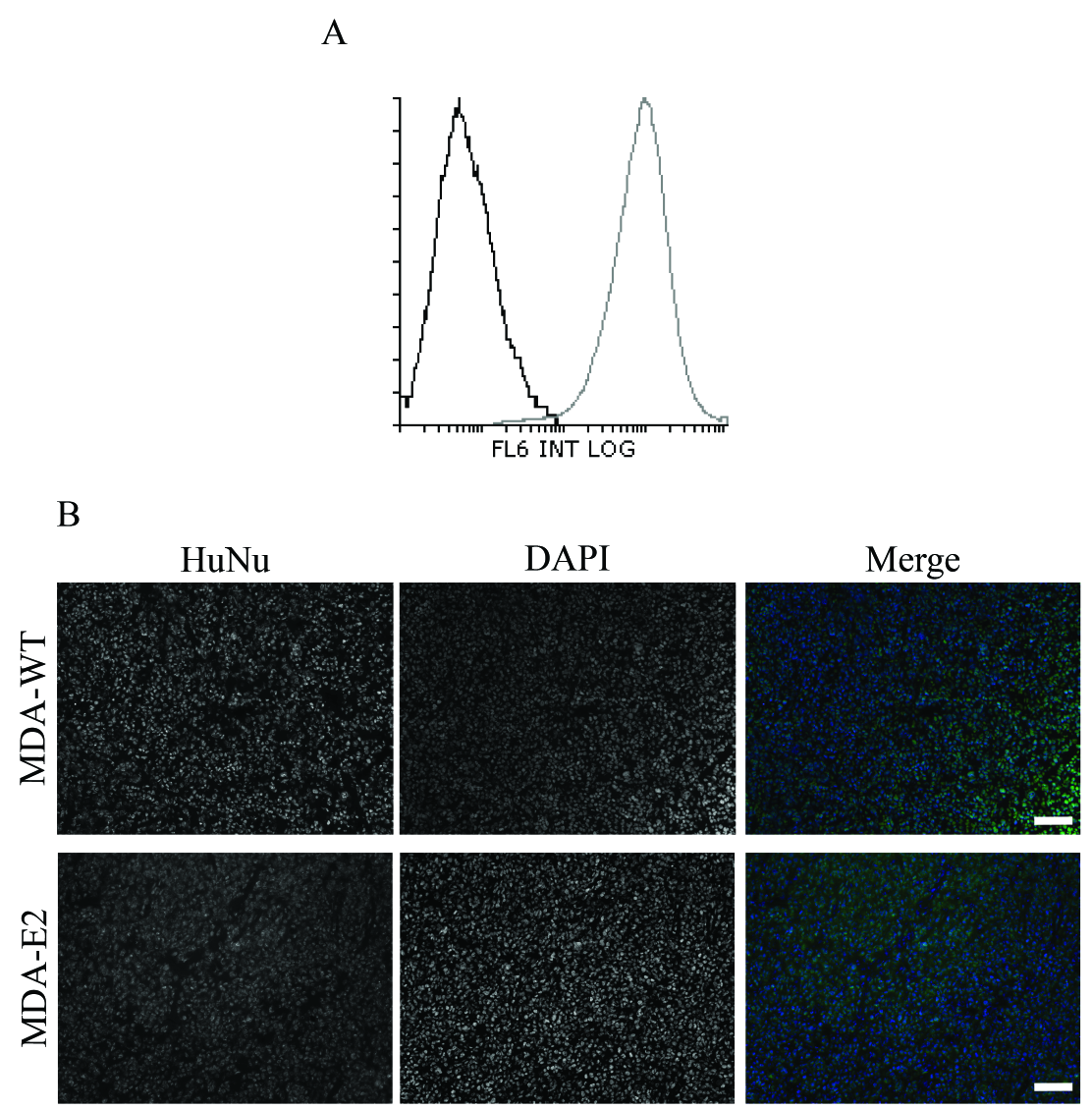

Supplement: S2 Fig — (A) Flow cytometry histogram of cells from dissociated tumor tissue from MDA-WT (black line) and MDA-E2 (gray line). (B) Representative histological image of MDA-E2 and MDA-WT tumors stained with antibody against human nuclei (green). The nuclei were stained with DAPI (blue). (TIF) [file pone.0132725.s002.tif]

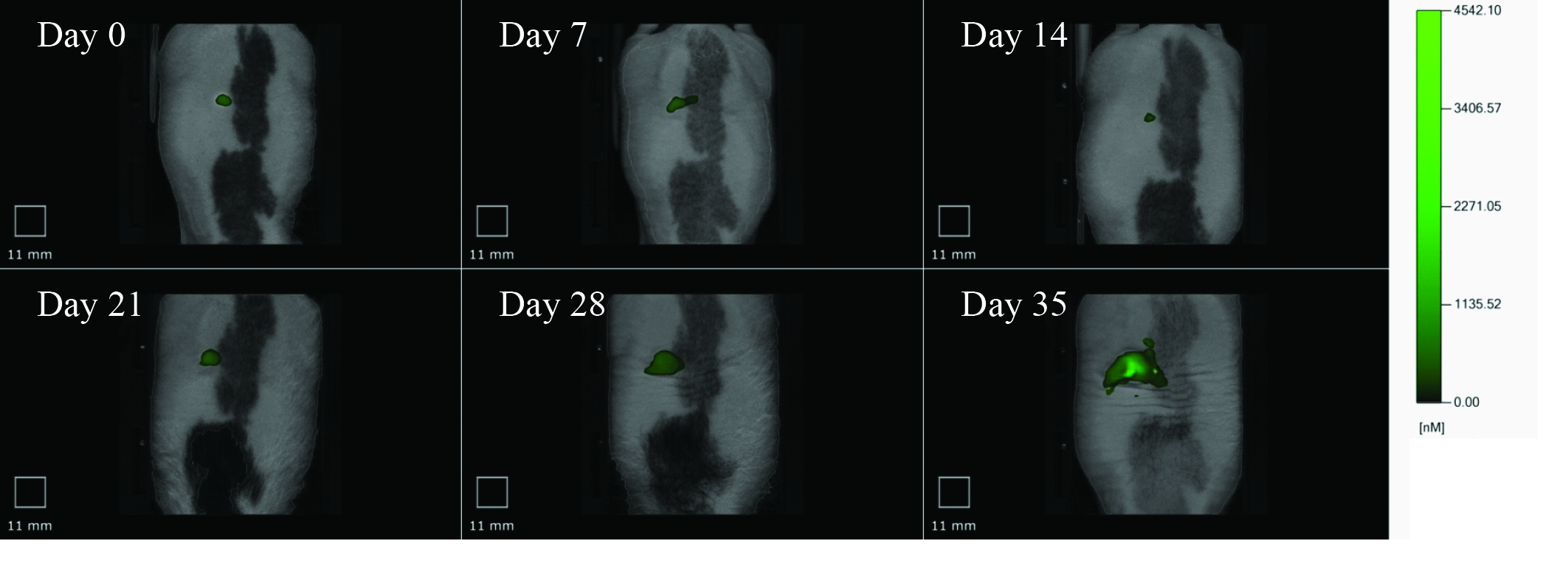

Supplement: S3 Fig — (A) Representative longitudinal image sequence of an animal bearing a MDA-E2 tumor. Images were taken at indicated times using FT. (TIF) [file pone.0132725.s003.tif]

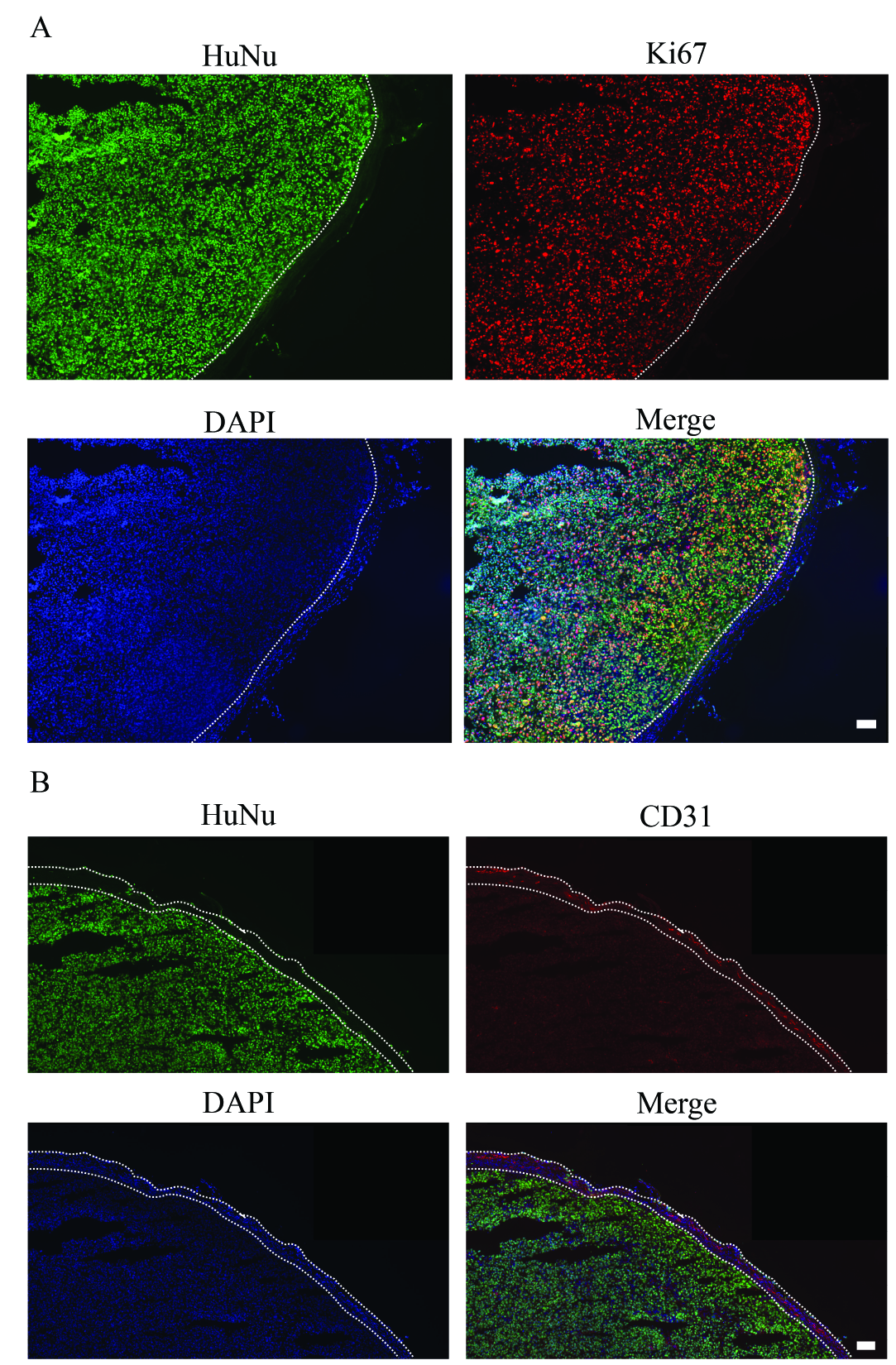

Supplement: S4 Fig — Representative histological images from MDA-E2 tumors. (A) MDA-E2 tumor stained with antibodies against CD31 (red) and human nuclei (green). The nuclei were stained with DAPI (blue). (B) MDA-E2 tumor stained with antibodies against Ki67 (red) and human nuclei (green). The nuclei were stained with DAPI (blue). (TIF) [file pone.0132725.s004.tif]
